# Preventive effect of methanolic extract of Zataria Multiflora Boiss on liver toxicity of paracetamol in rats

**Published:** 2015

**Authors:** A Ahmadipour, F Sharififar, A Najafi, J Atashbar, S Karami-Mohajeri

**Affiliations:** * Pharmaceutics Research Center, Kerman University of Medical Sciences, Kerman, Iran; ** Herbal and Traditional Medicines Research Center, Kerman University of Medical Sciences, Kerman, Iran; *** Physiology Research Center and Department of Physiology and Pharmacology, Kerman University of Medical Sciences, Iran

**Keywords:** Z. Multiflora, Paracetamol, hepatoprotective, liver toxicity, antioxidant activity

## Abstract

**Background:** The analgesic paracetamol causes a potentially fatal, centrilobular hepatic necrosis when taken in misuse and overdose. This research aimed to evaluate the protective effects of methanolic extract of Zataria Multiflora Boiss (Z. Multiflora) against hepatic damage induced by paracetamol-induced hepatotoxicity in male Wistar rats.

**Methods:** for this purpose, paracetamol was administrated orally at a dose of 2 g/ kg body weight (b.w.)/ day on the seventh day after the oral administration of a methanolic extract of Z. Multiflora at doses of 100 mg/ kg, 200 mg/ kg and 400 mg/ kg b.w. The lipid peroxidation level and activities of liver aminotransferases and enzymes contributing to the oxidative damage were measured in serum, and a histopathological examination of liver sections was also performed.

**Results and Discussion:** The results showed that Z. Multiflora reduced the activity of aminotransferases in rats treated with paracetamol. This extract also inhibited lipid peroxidation and protein carbonylation by an increase in the activity of the antioxidant enzyme and the elevation of glutathione content of the liver.

**Conclusion:** These effects are related to the antioxidant compounds of Z. Multiflora. The methanolic extract of this herb exhibits protective effects against paracetamol-induced hepatotoxicity.

## Introduction

Liver is an organ that has a significant role in the vital biochemical and physiological activities such as body homeostasis, growth, energy production, the supply of nutrients, and detoxification of drugs and other xenobiotics [**1**,**2**]. Therefore, liver is very susceptible to damage by hepatotoxic agents [**3**]. 

Paracetamol is an analgesic drug with a perfect safety profile in everyday use but in overdose, it can cause liver and kidney toxicity [**4**]. Paracetamol is activated by cytochrome P450 enzymes and produces N-acetyl-P-benzoquinone imine (NAPQI), a toxic metabolite that causes oxidative stress and glutathione (GSH) discharge [**5**].

The are many new drugs developed for the protection of the liver, but they have adverse side effects such as insomnia, vomiting, constipation, and depression [**6**,**7**]. Many existing plants are often used to treat a wide variety of clinical diseases including liver disease [**8**,**9**]. For this reason, further research on plants that have hepatoprotective properties and can be a safer alternative to chemical-based drugs, is critical [**8**]. 

Avishai Shirazi is the Persian name for Zataria Multiflora Boiss (Z. Multiflora), belonging to the family Labiatae [**10**]. There are too many studies on the antioxidant, antimicrobial, analgesic, and anti-inflammatory effects of this plant [**11**-**15**]. The phenolic compounds of methanolic extracts of Z. Multiflora are known to have good antioxidant activity [**12**]. Flavonoids and phenolic compounds in herbal extracts are free radicals scavengers and play the key role of antioxidants in the biological systems [**16**]. 

Our research survey revealed that no attempt has been made to this date to study the hepatoprotective activity of methanolic extracts of Z. Multiflora. Hence, we took this chance to study the hepatoprotective activity of methanolic extract of Z. Multiflora by using the paracetamol-induced liver damage model in rats. 

## Materials and methods

**Drug and chemicals**

Paracetamol and the other chemicals were obtained in high purity from Sigma-Aldrich Co., Ltd.

**Animals**

The study was made on adult male Wistar rats (200 ± 10gr) that were fed with standard feed pellets. All the animals were kept under conditions of controlled temperature (25 ± 2°C) and exposed to 12/ 12-hour light-dark cycle [**17**]. This research was approved by the Medical Ethics Committee of Kerman University of Medical Sciences (reference No.EC/KNR/89-13). 

**Preparation of plant extracts**

Fresh aerial parts of Z. Multiflora plants were dried at the room temperature and powdered. A sequential extraction of the leaf was carried out by using petroleum ether, chloroform, and methanol. Finally, the methanol extract evaporated at 45°C with a rotary evaporator, and the dry powder of the extract was collected and stored in a refrigerator at 4°C for further use.

**Animal treatment**

The animals were stochastically classified into five groups of eight rats in each group as it follows: (Group I) Normal mice received distilled water for a week. (Group II) Hepatotoxic bearing rats received an intraperitoneal (IP) single dose of paracetamol 200 mg/ kg body weight (b.w.). (Group III) Pretreatment group received the methanolic extract of Z. Multiflora (100 mg/ kg b.w., orally) for a week. (Group IV) Pretreatment group received the methanolic extract of Z. Multiflora (200 mg/ kg, orally) for a week. (Group V) Pretreatment group received Z. Multiflora (400 mg/ kg, orally) for a week. 

**Herbal preparation method**

After a one-week pretreatment with Z. Multiflora and 24 hours after the administration of one dose injection of paracetamol, all the rats were anesthetized with ketamine and xylazine [**18**]. Blood samples were collected by cardiac puncture and then centrifuged and plasma was separated from blood cells. The liver was also removed immediately, washed in cold saline, and homogenized in 0.2 M Phosphate buffer saline. All the samples were kept at a temperature of 80°C for further analysis.

**Histopathological Studies**

The liver was fixed in 10% formalin solution, and then embedded in paraffin wax by using conventional methods. Sections of 5 microns were stained with hematoxylin and eosin-stained and they were observed under a microscope for the evaluation of histopathological changes and for taking photomicrographs.

**Liver function tests**

Aspartate aminotransferase (AST) and colorimetric determination of alanine aminotransferase (ALT) were estimated according to the 2,4-dinitrophenylhydrazine method described by Reitman and Frankel [**19**]. The absorbance was determined at 546 nm by spectrophotometry.

**Oxidative stress markers**

Liver homogenates were used for the determination of lipid peroxidation (LPO) by a thiobarbituric acid reaction based method described by Ohkawa and et al. in 1979 [**20**]. Tissue nitrite (NO2-) and nitrate (NO3-) levels were calculated as an indicator of NO. Griess reaction that contained sulfanilamide and N-1-napthylethylenediamine dihydrochloride was used for measuring the total nitrite (nitrite + nitrate) at 545 nm after the conversion of nitrate to nitrite by copperized cadmium granules [**21**]. Finally, results were expressed as micromole/ gr of tissue.

**Enzymatic antioxidant status**

The homogenized liver was used in the determination of superoxide dismutase (SOD) [**22**], catalase (CAT) [**23**], and glutathione peroxidase (GPx) [**24**] activities.

**Statistical Analysis**

Results were expressed as mean ± standard error of mean (SEM) and analyzed by SPSS 16.0 software. The difference between the two groups investigated the one-way analysis of variance (ANOVA) followed by Dunnett’s test. The amount of P < 0.05 was considered statistically significant.

## Results

Liver histopathology and serum aminotransferase level

**Fig. 1 A-E:**
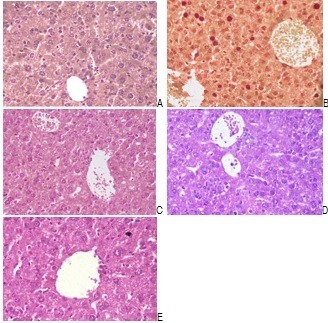
Healthy cells with round nuclei and eosinophilic cytoplasm separated by hepatic sinusoids

**Fig. 2:**
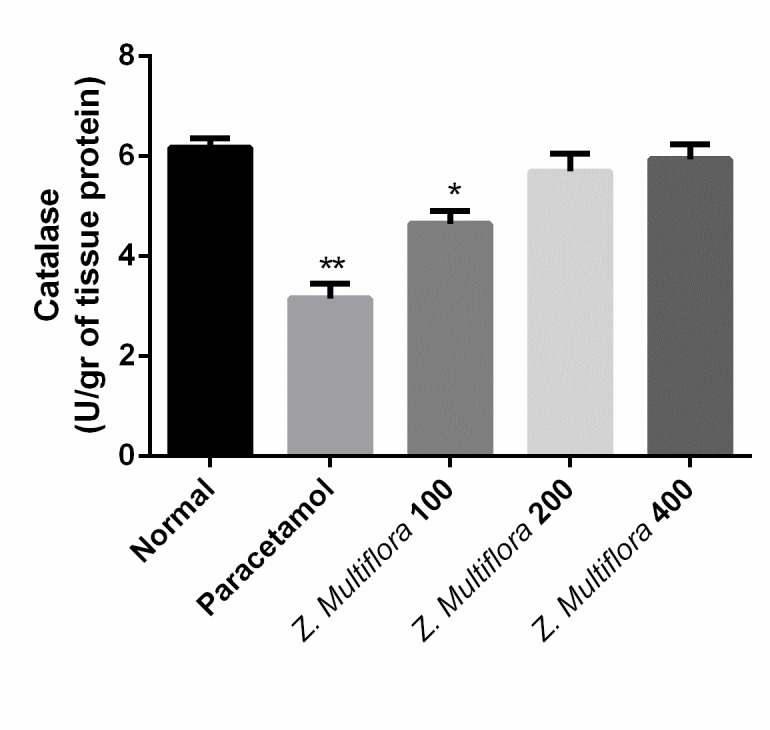
Photomicrographs of liver sections obtained from the normal group

**Fig. 3:**
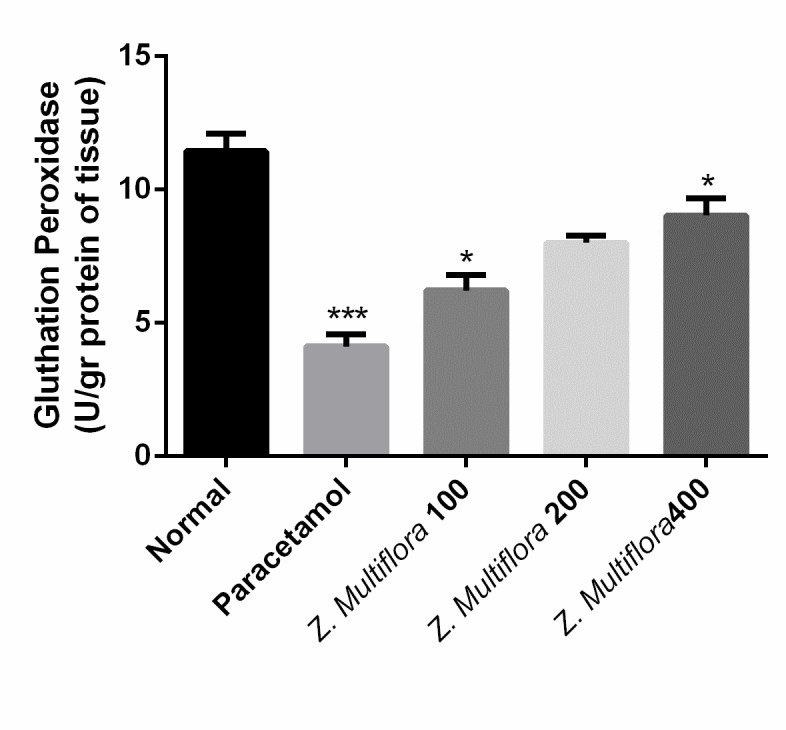
Paracetamol–induced group on the lipid peroxidation of liver tissue in comparison with positive and negative control groups

**Fig. 4:**
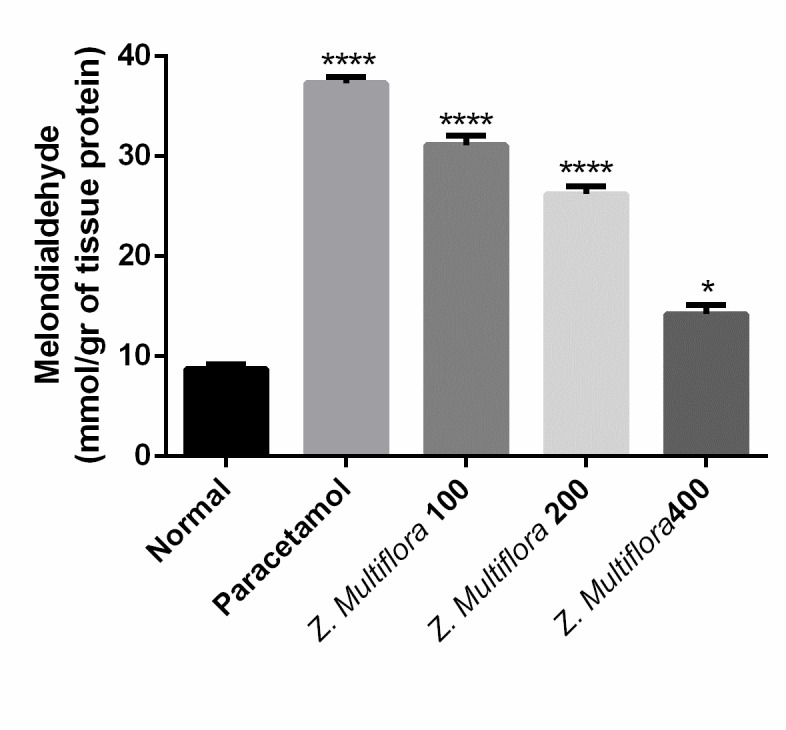
Effect of paracetamol + Z. Multiflora (100 mg/ kg) on the glutathione content of liver tissue in comparison with positive and negative control groups

**Fig. 5:**
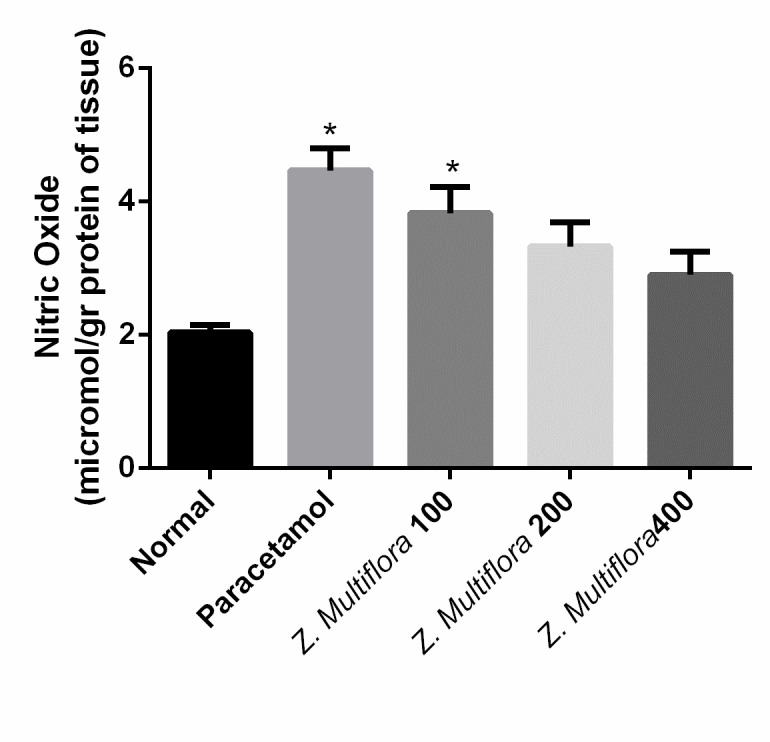
Effect of paracetamol + Z. Multiflora (200 mg/ kg) group on the nitric oxide content of liver tissue in comparison with positive and negative control groups

**Fig. 6:**
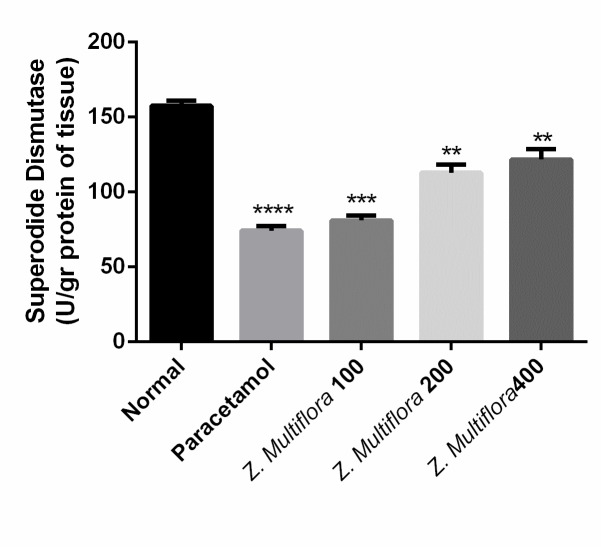
Effect of paracetamol + Z. Multiflora (400 mg/ kg) group on the activity of superoxide dismutase enzyme in comparison with positive and negative control groups

Light microscopic observation revealed healthy cells with round nuclei and eosinophilic cytoplasm separated by hepatic sinusoids (**Fig. 1A-E**). In contrast, groups receiving Paracetamol in doses of 200 ml/ kg b.w showed massive hepatotoxicity involved appearing from empty vacuoles aligned by strands of necrotic hepatocytes, the presence of dense focal inflammatory cells and many spots of focal cellular granulomatous lesions, and necrotic tissues (**Fig. 2**). Z. Multiflora methanolic extract exhibited reduced inflammatory cellular infiltration and hepatocytic damages. The hepatic tissues revealed the presence of thick focal inflammatory cells or necrotic tissues (**Fig. 3**-**6**). These histological abnormalities coincided with a significant increase in activity of ALT, AST, and ALP (**Table 1**); the treatment with Z. Multiflora methanolic extract significantly restored these levels to normal values (p < 0.001).

**Table 1:** Effect of various doses of Z. Multiflora on the activity of the serum aminotransferase enzymes in comparison with positive and negative control groups

	normal	cisplatin	cisplatin + Zm 100	cisplatin + Zm 200	cisplatin + Zm 400	Sig
AST	211.58 ± 17.99	289.85 ± 20.85	278.35 ± 11.39	252.98 ± 10.94	233.63 ± 9.69	p < 0.001
ALT	101.15 ± 7.89	188.83 ± 9.14	154.76 ± 8.86	129.65 ± 8.72	117.14 ± 6.71	p < 0.001
CAT	6.17 ± 0.45	3.15 ± 0.71	4.65 ± 0.61	5.69 ± 0.88	5.94 ± 0.73	p < 0.001
SOD	157.61 ± 8.54	74.26 ± 7.86	81.01 ± 8.18	112.98 ± 13.38	121.76 ± 16.75	p < 0.001
GSH	11.44 ± 1.57	4.11 ± 1.13	6.22 ± 1.41	8.01 ± 0.63	9.03 ± 1.58	p < 0.001
MDA	8.74 ± 1.71	37.32 ± 1.43	31.11 ± 2.29	26.23 ± 1.96	14.21 ± 2.240	p < 0.001
NO	2.03 ± 0.28	4.46 ± 0.82	3.82 ± 0.96	3.32 ± 0.88	2.90 ± 0.84	p < 0.001

**Oxidative stress biomarkers**

Paracetamol-intoxicated rats clearly showed a significant increase in the levels of MDA, SOD, CAT, NO, and GPx. Pretreatment with different doses of Z. Multiflora (100, 200, and 400 mg/ kg body weight) exhibited a significant reduction (P < 0.001) in MDA, SOD, CAT, and GPx. Rats treated individually with Z. Multiflora (400 mg/ kg of body weight) showed a near normal level in MDA, SOD, NO, CAT, and GPx compared to the control group.

**Statistical analysis**

Results were shown as mean ± Standard Error of Mean (SEM). The statistical significance of the differences between the groups was determined by ANOVA followed by the Posthoc Tukey test. Probability (P) value of less than 0.05 was taken to indicate the statistical significance.

## Discussion

The reactive oxygen species (ROS) are believed to be the mechanism of paracetamol-induced cellular injury. The balance between the oxidant and the antioxidant system seemed to be disturbed in our study by the paracetamol injection. In the present study, the high dose of paracetamol administration caused a hepatic lipid peroxidation and the consumption of antioxidant enzymes [**25**,**26**].

Also in the cur rent study, liver disorder was induced by paracetamol revealed by hepatocyte degeneration, inflammatory infiltration, and necrosis in liver tissue and by the elevation in the activities of serum ALT, ALP, and AST. The other findings obtained in this study indicated that the extract prepared of Z. Multiflora is effective against paracetamol-induced toxicity in the livers of male rats. This plant extract’s radical scavenging capacity can be used to justify its utilization in the therapy of various illnesses in traditional medicine. The drug profile of the extract in the in vivo investigations and clinical trials should be further investigated.

**Conclusion**

Finally, based on this research, we can conclude that the state of Z. Multiflora methanol causing a general protective effect against liver damage caused by paracetamol improved. The protective effect of Z. Multiflora is right about the content of methionine and antioxidant properties. It is likely to be restored by a free comprehensive collector, an inhibitor of lipid peroxidation and glutathione levels.
